# Evidence of cultural group selection in territorial lobstering in Maine

**DOI:** 10.1007/s11625-017-0501-x

**Published:** 2017-11-17

**Authors:** Timothy Waring, James Acheson

**Affiliations:** 1 School of Economics and Mitchell Center for Sustainability Solutions, University of Maine, Orono, ME 04469 USA; 2 Department of Anthropology and School of Marine Sciences, University of Maine, Orono, ME 04469 USA

**Keywords:** Cultural group selection, Lobster, Territoriality, Fisheries management, Cooperation, Cultural evolution

## Abstract

Relatively little is known about how resource conservation practices and institutions emerge. We examine the historical emergence of territoriality and conservation rules in Maine’s lobstering industry, using a cultural evolutionary perspective. Cultural evolution suggests that cultural adaptations such as practices and institutions arise as a result of evolutionary selection pressure. The cultural multilevel selection framework of Waring et al. (Ecol Soc, 2015) further proposes that group cultural adaptations tend to emerge at a level of social organization corresponding to the underlying dilemma. Drawing on detailed history and ethnography, we conduct a retrospective assessment to determine which levels of social organization experienced selection pressures that might explain the emergence of lobstering territoriality and conservation practices we observe in history. The evidence strongly suggests that informal territoriality evolved by selection on harbor gang behavior, while some conservation practices spread via selection at other levels from individuals to regional lobstering zones. We identify two apparent historical shifts in the dominant level of selection for these practices over the history of the industry and discuss the implications of this trajectory for the evolution of lobster management in the Gulf of Maine.

## Introduction

Practices and institutions that form a complementary ‘fit’ to the natural resource system they exploit are regarded as a central requirement for achieving environmental sustainability (Young 2002). The Maine lobster fishery is a classic example of institutional ‘fit’. The success of the Maine lobster fishery, in which catches have been high for decades (DMR 2015), is likely due to a combination of favorable ecological circumstances (Steneck et al. 2011) and conservation laws and practices especially tailored to lobster biology (Acheson 2003). However, the processes that create ‘fit’ are rarely studied. In this paper, we explore the processes that generate the ‘fit’ of lobstering practices and institutions to lobster populations. We propose that the theory of cultural evolution helps to explain the emergence of institutional ‘fit’ in the case of Maine lobstering.

The fields of environmental resource management and sustainability can gain added precision in understanding behavioral and institutional change by incorporating key aspects of cultural evolution (Boyd and Richerson 1985). Consider the process of institutional emergence. Cultural evolution describes how cultural traits such as behaviors, beliefs, and institutions become adapted to their social and natural environment (Richerson and Boyd 2005). People transmit cultural traits when they seek to improve their behavioral repertoires through imitation and teaching. Traits appearing to convey an advantage to those who hold them are often imitated more frequently than others, causing them to spread, while traits that appear less useful may tend to dwindle. The outcomes of newly adopted cultural traits, and the accumulated effects of imitation, result in novel cultural adaptations—behaviors and institutions that are particularly suited to their ecological, economic, or social environments. Thus, a ‘fit’ between institutions and the environment can be described as a special type of cultural adaptation (Hodgson 2010). But cultural evolution does not guarantee environmental sustainability or ‘fit’. So, to analyze social-ecological processes we must identify the forces that drive the evolution of culture.

Environmental fields can also benefit from the findings of cooperation research. The conservation of natural resources such as lobster usually entails a social dilemma—a situation in which the best individual outcomes (e.g., greater individual extraction) are at odds with the best group outcomes (e.g., sustainable extraction). In a social dilemma, achieving a group outcome such as sustainable extraction requires individual cooperation (Elster 1989; Taylor 1990). When conservation is a social dilemma, individuals must cooperate (e.g., by restraining harvesting effort) to achieve long-term resource viability. While the evolution of cooperation in social dilemmas is well understood, less is known about how cooperation and institutions evolve in real-world environmental social dilemmas. One general mechanism by which cooperation evolves is group selection (Field 2008; Nowak 2006). Group selection occurs when natural selection acts on groups instead of individuals. Intuitively, when players on a sports team compete with each other they develop strategies for individual superiority. But, when teams compete, teams with cooperative play and coordinated strategies tend to win, and those behaviors tend to be imitated by other teams and players. This process is known as “cultural group selection.” Thus, research on the evolution of culture and cooperation provides useful guides in understanding the origins and persistence of conservation behaviors.

In this article, we apply the cultural multilevel selection framework of Waring et al. (2015) to help explain the emergence of conservation-oriented behaviors and group territoriality in the Maine lobster industry.

## Lobster fishing in Maine

Lobsters are largely sedentary. Mature lobsters have an annual range of only 32 km (Campbell and Stasko 1986). Lobsters are caught with traps that rest on the ocean floor and are marked with a floating buoy. Legally, a state license is required to set lobster traps. But unofficially, one also needs to be accepted as a member of a harbor gang (Acheson 1988). Harbor gangs are lobster-fishing groups that defend an unofficial territory. Territories are defined by geographical features (e.g., coves, ledges, river mouths, buoys), and positioning systems including Loran and GPS, and are typically smaller than 100 square miles (Acheson 2003). As a result, lobster populations can be sustained and defended within these territories. Gangs defend their territories from intruders through verbal warnings and by molesting and destroying the lobster traps of an intruder. But molesting gear is against the law; and victims of trap cutting often retaliate. Thus, both defensive and offensive territorial behaviors carry significant risk.

Harbor gangs are typically small, with as few as eight boats and rarely as many as 50. Gangs are mostly composed of residents of a single harbor town, in which most members have long family histories. Harbor gangs are some of the most important social units in the lives of lobster fishermen.^1^ In times of need, members often turn to each other for help, and harbor gangs use a common radio channel in case of emergency. Harbor gangs are also somewhat socially isolated from each other, because harbor towns are separated by the jagged Maine coastline. People often hold negative stereotypes of people from neighboring harbors, and there are fewer friendly contacts than might be expected (Acheson 1988). Competition for lobsters and social status also occurs within harbor gangs. For instance, successful fishermen, or “highliners,” and senior fishermen typically enjoy more power, while poor fishermen or “dubs” are often the butt of jokes and gossip (Acheson 1988).

In the case of lobster, territorialism and conservation practices are economically complementary behaviors. An area is economically defendable if the benefits of ownership outweigh the costs of defense (Brown 1964; Dyson-Hudson and Smith 1978; King 1976). Lobsters can be spatially monopolized because they are relatively sedentary, and lobster territories can generate large profits, even when defending them is costly and risky. Conservation measures complement territoriality because the benefits of lobster conservation efforts accrue to those in control of the territory. Defended territory also enhances conservation efforts by limiting access and facilitating monitoring and enforcement. At first glance, neither conservation nor territoriality require an explanation of cooperation.

However, Maine lobster territories are claimed and defended by harbor gangs, not by individuals. Like any human group, gangs must overcome challenges such as coordination, within-group competition, and free-riding to survive and be successful. In comparison to unrestrained solo lobstering, gang membership can cause a reduction in harvest intensity and restrictions on harvesting location, and may entail risky territorial skirmishes. As a result, the present gang-based equilibrium may not be individually efficient. Moreover, we still need to explain how costly behaviors such as restrained harvesting and risky territorial defense emerged in the first place. We argue that group-structured cultural evolution helps explain how territoriality and conservation practices emerged, and why they persist. A brief historical summary provides an overview of the patterns we seek to explain.

### Preindustrial era (1700s–1900)

Before lobstering was widely practiced there was no benefit to exclusive ownership of lobster grounds. Prior to the twentieth century, lobstering was conducted from small, unpowered boats, making the range of each fisherman small. Most lobster fishing was done in the summer months when lobsters congregate near shore. These small near-shore areas were exploited by individuals or small groups of kin or neighbors; the areas were generally adjacent to property owned by the fishermen or their families. We call these “individual territories” although they involved more than a single fisherman in some instances. As lobstering became more widespread, fishermen came to benefit from protecting the water they fished, and small harbor group territories arose that were easy to guard and close to home. Although very little information exists on how the territorial system was first formed, it was commonly believed that ownership of land gave the landowner right to fish in adjacent waters.

### Informal era (1900–1995)

As lobstering became more common, the territorial competition between lobstermen in densely settled harbors grew increasingly fierce. Eventually, individual territories became unworkable, and fishermen started to cooperate to defend their territories from others. In the 1920s, engines allowed fishermen to range over more water, which allowed territorial growth and spurred conflict between neighboring harbor gangs. Conflicts close to harbors were fought bitterly and resolved with reinforced territorial boundaries. A pattern of harbor gangs protecting exclusive territories appears to have spread along the Maine coast and may share a common cultural lineage with a similar system found in nearby Nova Scotia (Wagner and Davis 2004). Some harbor gangs adopted perimeter defense, voluntary trap limits, and other practices that strengthened group structure. Island communities were often first to implement stronger conservation and territorial measures. These largely informal institutions heavily influenced how lobsters were managed in the twentieth century.

### Legal era (1995–present)

From the 1950s, most harbor gangs suffered from internal competition in the form of trap escalation. One solution to the problem was a limit on the number lobster traps a fisherman could set. Efforts to get the legislature to enact a trap limit law failed repeatedly due to disagreements on what the trap limit should be. In frustration, four islands established effective trap limits within their informal territories in the 1970s. Fishermen in different coastal regions preferred different trap limits because they faced different ecological (e.g., density of lobsters) and economic (e.g., distance to market) conditions. Thus, state-wide trap limit proposals failed for decades. In 1995, the Maine Lobster Zone Management Law created a legal territorial system for seven coastal regions, enforced by a professional warden force. The law resolved the 2-decade conflict over trap limits, and established Zone Councils with democratic structure and limited autonomy. Zone councils adopted policies including zone-specific trap limits and limited entry rules which have spread among most zones.

The proposal that changes in lobster fishing practice and policy constitute a process of cultural group selection leads to many questions that we cannot address here. However, the multilevel framework of Waring et al. (2015) allows us to ask new questions. Did today’s territorial and conservation practices emerge via selection among individuals, harbor gangs, or lobster zones? Has the dominant level of selection changed over time?

## Evidence of cultural group selection

Cultural group selection is a common and intuitive process, but collecting evidence of it requires careful accounting. Cultural group selection requires only two *necessary factors*: various (a) group cultural states which (b) spread between groups differentially. First, a *group cultural state* might be either an institution or a group-level aspect of individual behavior. Institutions, such as the presence of a voting rule, are often readily observable. But group-level differences in individual behavior, such as different average harvesting intensities between groups, are usually harder to detect. Second, group cultural states must spread differentially between groups. *Mechanisms of spread* include both social learning mechanisms (e.g., imitation) and natural selection (e.g., differential group survival) (Henrich 2004). Direct evidence of cultural transmission is rare outside of the laboratory, but the record of trait adoption among groups provides a reliable proxy. To illustrate, some observable traits (e.g., antennas on boats revealing radio use) might spread by imitation to neighboring gangs, while an unobservable trait (e.g., an effective bookkeeping system) might never be imitated yet still spread if groups that do not figure it out tend to expire, while those that do expand or multiply. These two necessary factors create the conditions for selection to act on group cultural states. If strong enough, cultural group selection can generate group-level cultural adaptations.

Cultural group selection is also intuitive because its signature outcomes are *group cultural adaptations*, which can be easy to identify.^2^ Group cultural adaptations can be institutions^3^ or collections of individual traits advantageous to the group.^4^ One individual trait that has special relevance for group selection is individual cooperation. *Individual cooperation*, costly individual behavior which benefits the group, is more likely to arise through cultural group selection than individual-level selection (Nowak 2006). Two primary factors influence the likelihood of group selection for a cooperative trait: (1) the ratio of trait variation found between groups versus individuals, and (2) the ratio of net benefits to groups versus individuals (e.g., see Bowles 2006; Rogers 1990). Other group cultural adaptations include behaviors and institutions that support individual cooperation. For example, individual *supporting behaviors* include peer monitoring, and punishment of non-cooperators, and *supporting institutions* include membership criteria, dispute resolution mechanisms, and collective choice rules, such as Ostrom’s design principles for group efficacy (Wilson et al. 2013). Table 1 lists types of evidence for between-group selection on cultural traits.

**Table 1:** An empirical rubric for organizing evidence of between-group cultural evolution

	Type of evidence	Item description
** Necessary factors**	Group cultural states	Institutions vary by group
		Individual behavior varies by group
	Mechanisms of spread	Social learning between groups (imitation, teaching, strategic migration)
		Natural selection on groups (extinction, growth, proliferation, obligate migration)
**Group cultural adaptations**	Individual cooperation	Individually costly, group beneficial behavior
	Supporting behaviors	Coordination between individuals
		Punishment of non-cooperators*
		Resource-specific usage rules*
		Monitoring of resource use*
		Social markers
	Supporting institutions	Social group boundaries, exclusive access*
		Resource boundaries*
		Collective choice procedures*
		Dispute resolution practices*
		Self-governance*

List represents most reliable signals of cultural group selection but is neither exhaustive nor exclusive. Traits with asterisks correspond to Ostrom’s design principles for group efficacy (Wilson et al. 2013)

For empirical investigations, it is important to note that, the output of cultural group selection (group cultural adaptations) may also serve as future input, like any evolving system. For example, an institutional rule such as group-exclusive resource access is likely to be the product of cultural group selection, but may also facilitate future group selection by binding the fortunes of group members more tightly to the outcomes of the group’s resource management choices. As a result, it is not always possible to differentiate between the factors that contribute to cultural group selection and products that result from it. Instead, we must tally patterns that are consistent or inconsistent with the action of cultural group selection generally.

In the two subsequent sections, we employ this rubric to categorize the ethnographic and historical evidence on the behaviors or institutions of lobster conservation and territoriality. The resulting tally facilitates an estimate of whether those traits were the results of adaptive cultural evolution, and if so at which level of organization.

## Territoriality

Using the empirical rubric in Table 1, we find substantial evidence for cultural group selection on lobstering territoriality behaviors among fishermen in harbor gangs. Fundamentally, harbor gangs are robust social units, and their importance is hard to overlook. First, territorial institutions and behavior clearly vary by group, and have likely spread between groups. Moreover, some territorial behaviors appear to be cooperative in nature, which decreases the likelihood of explanations based on individual-level mechanisms. Finally, we also observe multiple behaviors and institutions that support the costly cooperative actions involved in territoriality.

Principally, harbor gangs are special *group cultural structures* for harvesting lobster, and territorial strategies vary between gangs. Two basic types of informal lobstering territories exist (Acheson 1975, 1988, 2003). Most mainland harbors exhibit nucleated territoriality, in which the sense of ownership and strength of retaliation is strong close to the harbor, but grows weaker with increasing distance. The second type, perimeter-defended territory, is sharply defined with peripheral boundaries known to the yard. In these territories, mostly observed around island harbors and a few mainland harbors in Penobscot Bay, the sense of ownership remains strong to the outer boundary, and the entire territory is strongly defended.

Although we have found no direct evidence of *strategic imitation*, social learning, or other mechanisms of spread for harbor gang tactics, it is extremely likely that harbor gangs learn from each other, and that territorial practices have spread between gangs as each gang seeks to adopt the best strategy for its environment.

Territorial strategy clearly influences *group outcomes*, and possibly group survival. Territorial defense and offense are both costly, and therefore engaging in too much of either can have negative consequences for a gang. First, invasions often fail (Acheson 2003, p. 35). The decisions to invade or defend depend on the fishing value of the area, the ability to monitor it, and the costs of transportation (Acheson and Gardner 2004, pp. 39–40). Defense is also risky. Typical gang defensive strategies such as cutting the lines of the invading traps are illegal, and risk personal violence and prosecution. Despite these costs, offensive and defensive actions do occur. Indeed, *certain strategies are more successful,* and may be selected for across gangs. For example, around 1960 fishermen from upper Muscongus Bay began fishing at the mouth of the bay in the fall and winter, throwing them into conflict with fishermen from New Harbor and Round Pond. Years of retaliatory trap cutting and hostility followed. In the end, however, the upper bay fishermen were able to invade the outer bay gradually, turning it into a mixed fishing area (Acheson 2003, pp. 43–44). This shows that lobstering territory can indeed be taken by force by a gang with aggressive expansion norms, benefiting them at a major loss for the defenders. This change in group-level outcomes is an example of *group-level natural selection*.

Sharing harvesting territory with other fishermen also may constitute *cooperative behavior*. Ordinarily, members of harbor gangs subdivide the territory “owned” by that gang and place traps to avoid areas used by fellow gang members. This requires *coordination*. However, some locations within a territory are better than others, being closer to harbor, more productive, or easier to navigate. Thus, there exists a possibility of internal competition, and to divide the area fairly fishermen must *cooperate*.

Participation in gang territorial actions clearly involves *cooperative behavior*. Territorial action is individually costly, but beneficial to the group. The benefits of capturing new territory, including more or better fishing area, more catches per trap, and fewer gear tangles are shared among members of the invading gang. However, individuals must bear the costs of invasion or defense, potentially including bodily harm, loss of traps, and the possibility of prosecution if caught. However, these costs are not shared equally. Territorial actions are usually conducted by small well-organized subgroups within a gang. It should be noted that, older men with high prestige often leave the risky actions to younger, “wilder” members, and get differential access to preferred lobster areas.

Harbor gangs also display many *behaviors and institutions to support* the costly acts of territorialism. For example, gangs exert *control over membership*, keeping the benefits of cooperation within the gang (Acheson 2003, pp. 30–33). Entry is more likely to be granted to those who come from a local family, have relatives and experience in the lobster business, who began fishing as a teenager with a few traps, and who are willing to support local norms. Newcomers with non-fishing income, who begin fishing with many traps, will be treated more roughly, and may not be accepted.

The *social limits to gang entry* also vary between gangs. For instance, joining perimeter-defended gangs is much more difficult than joining nucleated gangs (Acheson 1988). By the nature of their geography, island harbor gangs are typically smaller and more socially cohesive than those in mainland nucleated gangs. While most nucleated harbor gangs have members who were once outsiders, island gangs typically have more restrictive membership terms. In some island gangs, family ownership of island land is required (Acheson 2003, pp. 29–33).

Another *supporting institution* is informal processes of *collective decision-making*. Island gang members are known to communicate frequently and come to a consensus on a joint strategy for defense. At least two island gangs hold frequent formal meetings (Acheson 2003). Among other matters, such meetings decide who is going to repel the intruders. Table 2 summarizes the evidence supporting gang-structured cultural evolution in territorial behavior.

**Table 2:** Evidence for gang-level selection on informal territorial behaviors and institutions in Maine Lobster fishing

	Type of evidence	Item	Evidence pattern
**Necessary factors**	Group cultural states	Institutions vary by group	Territorial strategies vary between gangs. (Strategies: perimeter and nucleated. Only islands have perimeter defense)
		Behavior varies by group	Social cohesion varies between gangs. (Islands have greater cohesion)
			Social isolation between gangs leads to group-level variation. (Inter-group migration rare)
	Mechanisms of spread	Social learning between groups	*Similar strategies and customs appearing in many harbor gangs can be attributed to both independent invention and transmission*
		Natural selection on groups	Territorialism shapes gang payoffs. (Invasion of Lower Muscongus Bay)
			Group strategies differ in efficacy (nucleated easier to invade)
**Group cultural adaptations**	Individual cooperation	Cooperative behaviors	Individual costs for group benefits of territorial action (legal action, retaliation, violence)
		Greater group benefits	Group benefits from claiming, protecting, and exclusively harvesting a shared territory
		Lesser individual costs	Gang members support each other in times of need (indirect reciprocity)
			Large costs (e.g., violence) are intentionally risked—revealing high benefits at stake
			Small group size makes cooperation more likely (e.g., islands, mission teams)
	Supporting behaviors	Coordinated behaviors	Members coordinate (radio, in-gang trap placement)
		Monitoring of resource use	
		Social markers	Gang members share an exclusive social identity
	Supporting institutions	Social organization	Gangs have social organization and complex territorial strategies
		Social boundaries	Gangs limit membership by social proximity (harbor town, island)
		Resource boundaries	Gangs hold exclusive lobstering territory
		Collective choice	Frequent meetings to decide who repels intruders
		Dispute resolution	
		Self-governance	Gangs are informal, self-organized, self-governing

Indirect evidence is presented in italics

To summarize, harbor gangs are small and insular groups of lobstermen who share a strong social identity, and display a high degree of solidarity. Individuals also compete within gangs for lobster, space, and status. Gangs protect a territory, limiting access to a small number of fishermen, reducing trap competition. The act of defending lobstering territories entails individual risk, but generates group benefits. The risks of defense actions such as trap cutting are typically shared by multiple gang members. Perimeter defense is costlier to maintain than nucleated territory. And, gangs with perimeter defended territories are smaller, more isolated, more insular and more socially cohesive than most nucleated gangs. Thus, the informal territorial system has the necessary factors for cultural group selection, and displays many group cultural adaptations at the gang-level. Next, we conduct the same exercise for lobster conservation laws and informal conservation behaviors.

## Conservation

The Maine lobstering industry has a unique set of informal conservation norms and special conservation laws. For example, people taking undersized lobsters or reproductive females will face trouble from other fishermen as well as the state warden force (Acheson 2003, p. 17). To explain how practices such as voluntary peer monitoring and unique conservations laws came to be, we review and compare the six most significant conservation practices and policies in Maine’s lobstering history. Unlike territoriality, we do not always find evidence for cultural group selection on harbor gang behavior. Instead, some laws and practices seem to be spread via individual-level processes, while others appear to be the result of selection on entire lobstering regions. First, we examine three state lobster conservation laws.

### Double gauge law

The double gauge law establishes a maximum and a minimum size for saleable lobsters. Restrictions on allowable lobster size pose a *social dilemma*: while individuals who take all sizes will benefit, the size restrictions protect lobster reproduction and *benefit the industry*. The law was first proposed in 1905 for lobster conservation and industry longevity. But, *between group differences* caused by varying ecological and economic environments along the coast halted the law’s passage. Fishermen from western counties supported a smaller maximum gauge; those from the central and eastern coast were generally opposed. During the 1920 s and 1930 s the lobster industry suffered greatly from a dramatic decline in catches in 1919, and low prices caused by competition from Canadian lobsters the US economic depression of the 1920s. Nearly a third (32%) of lobstermen left the industry between 1928 and 1932—a type of cultural *natural selection* on fishermen. These crises contributed to the passage of the double gauge law in 1933 (Acheson 2003, pp. 85–88). In the early 1930s, the commissioner of marine resources and others *spread support* amongst fisherman and legislators, arguing the law would conserve the breeding stock and increase sales. Thus, the double gauge law appears to be a state-level collective-action adaptation to a difficult social dilemma. Individuals and harbor gangs were unwilling to adopt the practice voluntarily, and coastal regions disagreed. Thus, selection at the individual (against) and state levels (for) were in conflict. Political deadlock reigned until a major state-wide crisis (strong group-level selection) allowed the law to pass.

### V-notch law and custom

The V-notch law of 1948 bans the collection and sale of lobsters with a V-shaped notch cut into the right tail flipper. Creating such a mark is a *low cost individual action*. The law built on a prior 1917 law in which wardens bought egged females from lobster pounds, marked their tails and released them to improve population growth—*a state-level benefit*. Both laws prohibit fishermen from taking lobsters with marked tails. And, after World War II some fishermen took advantage of this law and began to mark egged lobsters they caught in the wild to preserve them for future breeding. This voluntary and easy practice of V-notching *spread amongst individual lobstermen* over a long period and has since become a widely held informal conservation norm (Acheson 2003, pp. 88–90). In this case, there were very few costs to modifying an existing regulation and no apparent social dilemma. Meanwhile, the low-cost practice of V-notching was adopted voluntarily. Thus, individual selection for the practice appears to have been sufficiently positive to support its adoption without state-level selection.

### Escape-vent law

The escape-vent law of 1978 requires that lobster traps include a small escape hole through which under-sized lobsters can escape. Some fishermen experimenting with these additional holes no longer had to throw back small lobsters and reaped such an *individual time and cost savings* that they had kept the innovation a secret. But *word got out*. In addition, Massachusetts and Newfoundland already had escape vent laws in place, which suggests that *state-level social learning* may have taken place. Thus, escape vents appear to provide enough individual benefit to explain the adoption of the trait without groups. The law received one of the easiest passages in the history of the industry (Acheson 2003, pp. 90–91). Therefore, in contrast to the double gauge requirement, installing escape vents carries individual economic benefits as well as group benefits, and poses *no social dilemma*. The practice spread individually, and the law was passed rapidly. Here again, it appears that selection at the individual and group level coincided, and were both positive.

Superficially, these three state laws can be considered state-level adaptations which conserved lobster populations and bolstered the industry in the long term. However, the emergence of these laws—the social process by which the necessary political support accumulated—differs. The main difference is that the double gauge measure posed a social dilemma amongst fishermen across the state while the other two laws did not. Because of this fact, the double gauge law was contentious, and its passage appears to have required a major state-wide industrial catastrophe to galvanize the sufficient support. By contrast, the other two laws seem driven by individual mechanisms. The V-notching practice was nearly costless for individuals and escape vents offer clear individual benefits. These cases, summarized in Table 3, reveal the necessity of structured comparative analysis.

**Table 3:** Evidence for selection on lobster conservation practices across four levels of social organization

	Type of evidence	Law/practice: social scale of evidence	Double gauge	V-notching	Escape vents	Trap limits	Special zones	Lobster zones
**Necessary factors**	Group cultural states	Social scale of institutional variation	X	X	X	G	G	R
		Social scale of behavioral variation	X	X	X		G	R
	Mechanisms of spread	Scale of social learning		X	S/I	–	–	–
		Scale of natural selection (competition)	S			G	G	R
**Group cultural adaptations**	Individual cooperation	Scale of cooperation (social dilemma)	S	X	X	G	G	R
	Supporting behaviors	Individual coordination	✓	✓	X	✓	✓	✓
		Resource-specific usage rules	✓	✓	✓	✓	✓	✓
		Punishment of non-cooperators				–		
		Monitoring of resource use				G	G	R
		Social markers				G	G	
	Supporting institutions	Social group boundaries				G	G	R
		Resource boundaries				G	G	R
		Collective choice procedures				G		R
		Dispute resolution practices						
		Self-governance				G	G	R
**Selection on:**	Individuals		−	+	+	−	−	−
	Gangs					+	+	
	Regions							+
	State		+					
**Dominant Level of Selction:**			S	I	I	G	G	R

Evidence for selection at a given level is denoted with the letter representing that level; individual (I), gang (G), region (R), and state (S). Evidence for group-selection generally is denoted with a check mark, evidence against with X. The direction of selection at each level is denoted as positive (+) or negative (−)

### Voluntary trap limits

**Fig. 1:**
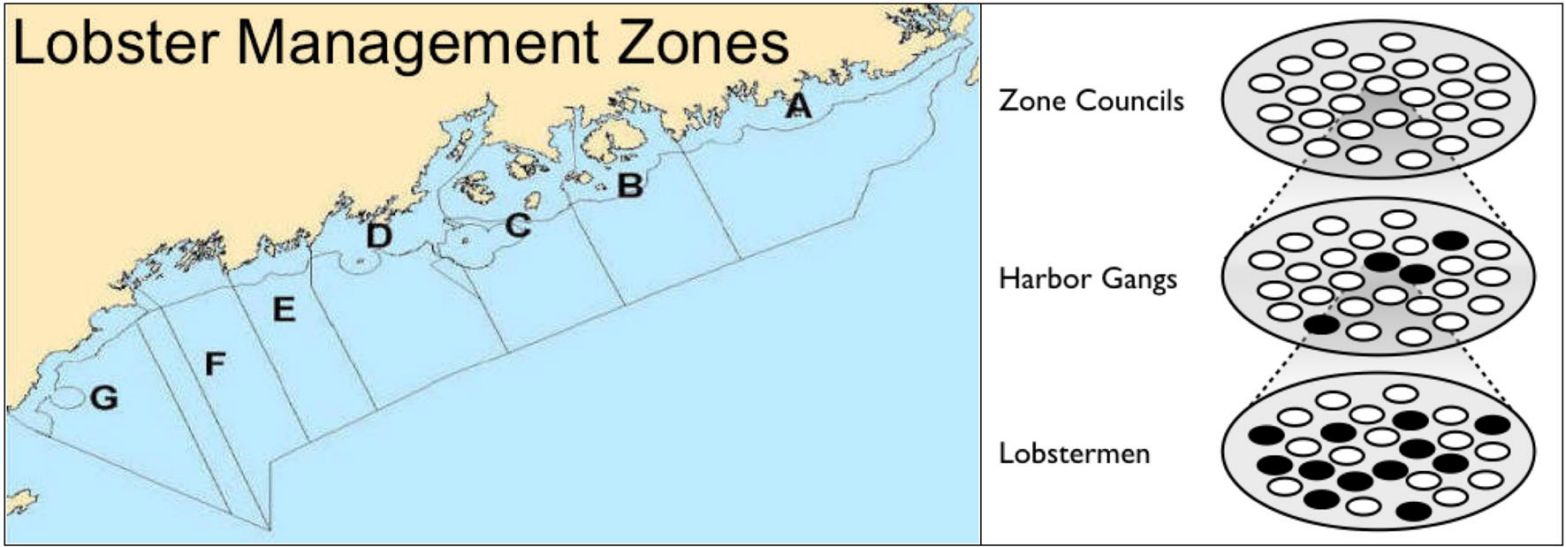
Seven lobster management zones were created. Note the buffer zones at the boundaries of Zones F/G, C/D, and E/D (not visible at this scale) (DMR 2003). White and black ovals represent individuals or social units for and against trap limits, respectively

Trap escalation poses a *quintessential social dilemma*. When gang members increase their trap numbers to achieve greater catches, and others follow suit, the result is a tragedy of the commons with fewer lobsters per trap and more work, congestion, and gear tangles. Since the 1950 s many lobstermen have favored trap limits. But while fishermen in the northeast preferred a 400-trap limit, those in the south sought a limit of 1600 traps (see map in Fig. 1). These *between-group differences* halted the passage of proposed trap limit bills in the legislature for 17 consecutive legislatures (Acheson 2003). However, informal trap limits *spread among harbor gangs* anyway. Starting in the 1970s, four island gangs (Monhegan, Criehaven, Swan’s and Green islands), frustrated with the lack of action at the state level, enacted local rules limiting the number of traps their fishermen could use in their unofficial territories, cutting costs for their fishermen (Acheson 2003, 57ff). These gangs shared a common prerequisite for motivating cooperation: tight-knit communities with stable membership, strong social bonds and a high degree of *social capital* (Singleton and Taylor 1992). In such communities, people have a set of *reciprocal relationships* and know who to trust. All four islands were already perimeter-defended. Moreover, three islands with *smaller gangs found it easier* to establish trap limits, while Swan’s Island had a larger number of fishermen and more trouble coordinating the efforts. In summary, when state trap limits failed to solve the collective action problem, harbor gangs with sufficiently strong group loyalty enacted unofficial voluntary trap limits for their own, group-level benefits. However, as should be expected of cooperative institutions, only harbor gangs with high social capital, were able to self-impose trap limits, while the larger, less cohesive and less isolated gangs could not.

### Special conservation zones

The ability of island gangs to enact group-beneficial limits on lobster harvest can also be seen in the first transitions from informal to legal territories. In 1984, Swan’s Island was experiencing the *social dilemma of trap escalation* and increasing *competition from mainland fishermen* seeking additional fishing grounds (Acheson 2003, pp. 50–51). Fishermen of Swan’s Island lobbied for and won special consideration from the Commissioner of Marine Resources, who converted the informal territory into an official lobster conservation zone. This regulation *strengthened their group territorial claim*, forbidding mainland fishermen from fishing the area, and established a very low trap limit, *solving their internal trap escalation* and forcing island fishermen to conserve lobsters. There is also good evidence that the 475-trap limit which Swan’s Island adopted cut costs and resulted in higher incomes. (see Acheson 2003).

A nearly identical process occurred on Monhegan Island. Incursions of mainland fishermen into the unofficial Monhegan territory caused *major group conflict* in which a lot of gear was destroyed and one Monhegan boat was sunk. Monhegan fishermen successfully lobbied the legislature for special conservation status around their island. In 1998, the new law imposed special rules for the Monhegan waters including a strict trap limit, an apprenticeship program, and a short fishing season. It also forbid boats from other harbors from fishing within two miles of Monhegan, officially *protecting their hitherto informal territory* (Acheson 2003). Finally, in 2002, Isle au Haut fishermen *observed the beneficial effects* of these two conservation zones and approached the legislature to have a similar zone established around their island. Although they were unsuccessful, the Isle au Haut attempt reveals *strategic imitation* of group-level policy. Thus, island lobster gangs *imitated each other* to obtain legal state protection and enforcement for their territories and solve the *internal cooperation problem* of trap escalation. Here again, small island communities with *high social capital*, interdependence and a *common fate* were the first to achieve this level of protection. It is significant that, to this day, the only harbor gangs granted such special dispensation are islands.

### Lobster management zones

The Lobster Zone Management Law (Public Law 495, Chap. 468) is the most significant law in the history of the industry. Passed in 1995, the law established a state-wide legal territorial system by dividing the coast into seven management zones, resolving the *conflict over trap limits* in different regions (Fig. 1). The law also established a statewide trap limit of 1200; a tag system to identify trap owners; an apprenticeship program; and lobster license eligibility requirements. But most important, the law gave zones significant *autonomy to self-govern* through limited management capacities of new zone councils (Acheson 2003, 103ff). Zone councils, elected by the lobster license holders of a zone, can propose rules on a small set of issues: trap limits; season timing; the number of traps per line, and entry limits. A proposed rule that garners a two-thirds vote automatically becomes a Department of Marine Resources (DMR) regulation and is enforced by state wardens. Lobstermen could now *collectively decide* on trap limits by region. In 3 years, all zones had voted in trap limits of between 600 and 800 traps, *reducing competition for lobsters within zones*. Zone councils also have the authority to propose *limits on entry* of fishermen into their zone. The limited entry provision *proliferated among zones*, and today all but zone C have limited entry rules. However, *territorial conflict between zones* also emerged. The new zones came into conflict about how many traps fishermen could place in a neighboring zone. A DMR rule stipulating that no more than 49% of a fisherman’s traps could be placed in another zone resolved only some of these conflicts (Acheson 2003, p. 112). In zones D and E, this solution was regarded as unfair because the trap limit of one’s zone influenced how many traps a fisherman could put in a neighboring zone. The commissioner ultimately resolved these conflicts by creating official buffer zones, in which fishermen from both zones would be allowed to fish, *reducing competition between zones*.

Thus, although the 1995 law was an act of the state, it devolved power to a new level of lobster management with limited self-governance power. Lobster zones used their new autonomy to achieve collective goals, resolving internal conflicts with trap limits, bolstering cooperation by limiting entry of new fishermen, and engaging in and sometimes resolving conflicts with neighboring zones. Trap limits and limited entry are examples of conservation measures that proved too costly for individual fishermen and even many harbor gangs to implement, but that most zones have nonetheless adopted, with beneficial zone-wide outcomes. It appears that rules such as the limited entry provision have been imitated between zones. Therefore, regional zones appear to be an important level of cultural selection in the lobster industry.

The six lobster conservation cases presented above can be compared by the organizational level implicated in each component of the framework (Table 3). In the cases of V-notches and escape vents, we mostly observed signs of individual-level cultural evolutionary forces, and find little to no indication of social dilemmas in either context. We do observe social dilemmas in the remaining cases. For voluntary trap limits and special conservation zones, we see evidence that a gang-level cultural adaptation emerged to solve a gang-level social dilemma. The history shows that harbor gangs played a decisive role in the spread of limited entry practices, informal trap limits, and special conservation zones with island gangs adopting these practices early and others gangs following suit. These practices bear signatures of group-level adaptations in that they reduce in-group competition and facilitate gang-level resource efficiency. Lobster management zones also appear to be a regional-level cultural adaptation to a regional-level dilemma, namely the problem of establishing trap limits. Finally, the double gauge requirement may have been a state-wide social dilemma which was only resolved when after a state-wide crisis and selection event increased the stakes. Table 3 also presents an estimate of the direction of selection on each practice for all levels, and an estimate of the dominant level of selection.

In summary, by gleaning evidence relating to group-level cultural adaptation and especially patterns of cooperation, we surmise that there are at least four levels of social organization which may experience cultural selection relative to lobster harvesting and conservation practice: individual, gang, zone, and state. Importantly, selection at the regional level, and the zone adaptations that it generated, only emerged in the mid 1990s. Thus, it is reasonable to conclude that solutions to the major conservation dilemmas in the Maine lobster industry in the last century have emerged via selection on groups closest to the social scale of those dilemmas. This finding may be applicable in other contexts, as well. That is, without external influence, conservation problems rooted in social dilemmas tend to be resolved by groups at the corresponding level of social organization, and therefore solutions only emerge as fast as the relevant groups evolve culturally.

## Evolutionary changes in lobster fishing practice

**Fig. 2:**
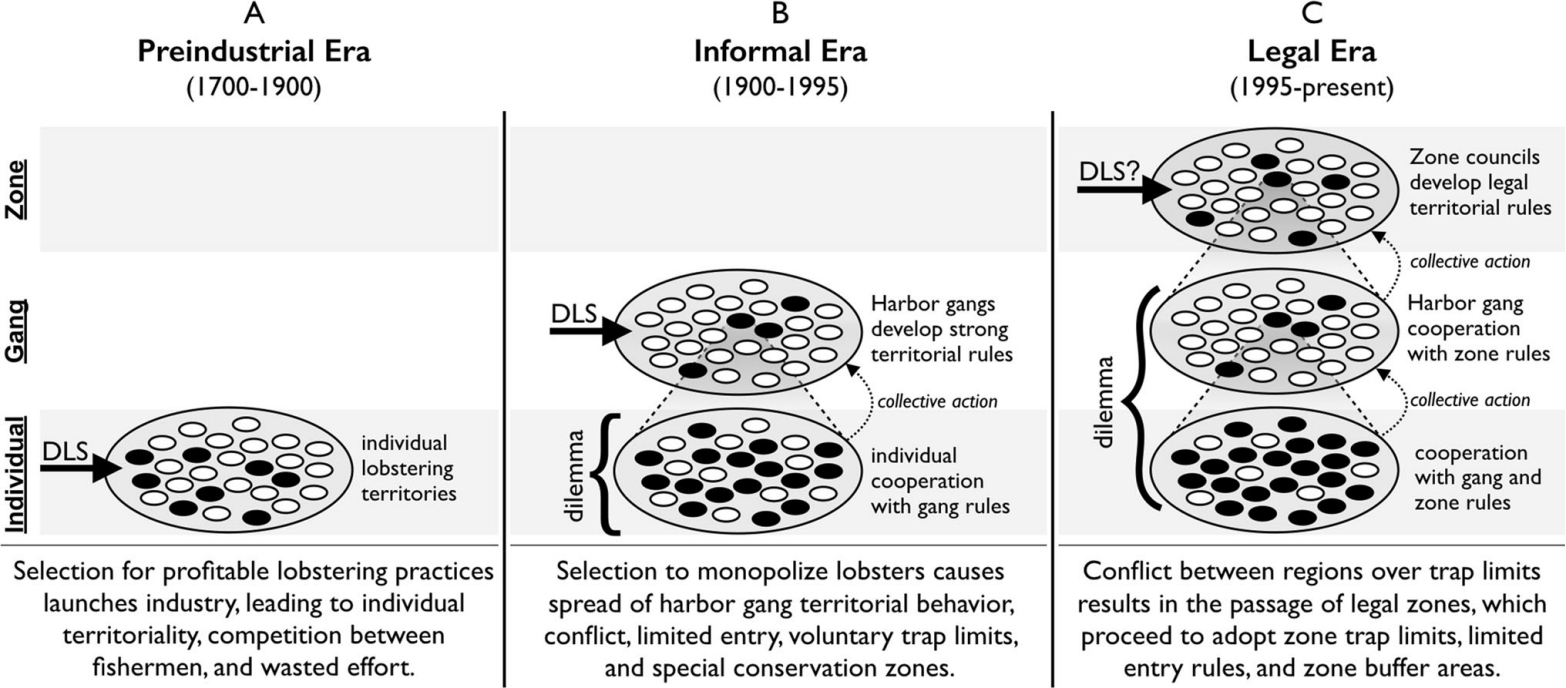
Likely changes in the level of selection for lobstering practice and policy (ca. 1700–present). *DLS* refers to the hypothesized dominant level of selection. White and black ovals represent social units with cooperative and non-cooperative cultural traits, respectively. The level of the state of Maine is not represented

Integrating the territorial and conservation cases, we can synthesize an evolutionary history of Maine lobster fishing practice and institutions. Historical evidence implicates group-structured cultural evolution at multiple levels (Table 4) and reveals changes in the dominant level of cultural adaptation over time (Fig. 2).

**Table 4:** Timeline of cultural adaptations in the lobstering industry

Date	Practice	Level	Adaptive problem
c1900	Harbor gangs emerge	Gang	Limit competition between lobstermen
c1900	Exclusive membership	Gang	Limit competition in gangs
1933	Double gauge law	Individual/state	Conserve lobsters locally
1948	V-notching practice & law	Individual/state	Conserve lobsters locally
1970s	4 islands enact trap limits	Gang	Limit trap escalation in gangs
1978	Escape vent law	Individual/state	Conserve lobsters locally
1984	Swan’s island conservation area	Gang	Limit trap escalation, conserve lobsters
1995	Zone management Law	Zone	New organizational level emerges
1998	Monhegan island conservation area	Gang	Limit trap escalation, conserve lobsters
1998	Zones adopt trap limits	Zone	Limit competition within zones
2000	4 zones pass limited entry	Zone	Limit competition within zones
2000s	Buffer areas between zones	Zone/state	Limit competition between zones
2000s	2 zones pass limited entry	Zone	Limit competition within zones

Lobstering territoriality and conservation practices constitute adaptations that solve adaptive problems at different organizational levels

For most of the twentieth century, harbor gangs appear to be the most active level of cultural adaptation in lobstering practice. Harbor gangs have significant autonomy and scope for collective action and they compete vigorously. They vary in size, profitability, and collective solidarity, and they share an economic problem which favors group solutions. Although harbor gangs do not often expire or proliferate, they do learn from each other, and successful practices have clearly spread between gangs. Then, starting in 1995, lobster management zones emerge and begin a process of rapid cultural adaptation. Zones are democratically self-governing with the autonomy to pass legally binding rules in a few arenas. Zones also face the shared economic problems associated with maintaining lobster stock and reducing competition between lobstermen. And, zones have adopted a series of successful conservation practices including trap limits and limited entry provisions, both of which have spread between zones, and both of which were originally traits of lobster gangs. Therefore, cultural adaptation relevant to lobster conservation appears to have occurred at both gang and zone levels. In addition, evidence suggests that some features of today’s lobster conservation system resulted from cultural evolution at the individual (escape vent), and state (double gauge) levels.

Table 4 reveals that the primary periods of adaptation for harbor gangs (majority of twentieth century) and lobster zones (1990s-present) are relatively non-overlapping, signaling that adaptations have slowed for harbor gangs and increased for zone councils.

We argue that two major transitions can be distinguished in the evolution of Maine lobstering practice (Fig. 2). The first shift, we speculate, was from individual fishermen or fishing families to harbor gangs. As competition for lobsters increased, so did the need to protect lobstering territory. Gangs became more effective holders of territory than individuals, and innovative rules and cooperative behaviors spread between gangs. Conflict between harbor gangs led to ever stronger territorial practices, such as perimeter defense and exclusive legal conservation zones, as well as institutional innovations such as trap limits that reduce in-group competition. Between-gang competition and gang-level institutional evolution drove lobstering practices for most of the twentieth century. The dominant level of selection for lobstering behavior had therefore changed from individual to gang. This interpretation is consistent with prior game-theoretical analysis (Acheson and Gardner 2005).

A second shift in the dominant level of selection seems to have occurred in response to the social dilemma of lobster trap limits (Fig. 2). Because only the most tight-knit island gangs were able to enact voluntary trap limits, within-gang competition via trap escalation remained a major problem. The possibility of official enforcement of trap limits was enticing but required between-gang cooperation. A state-wide limit was politically impossible because regions favored different trap limits. These pressures led to the creation of a new level of (legal) social organization between the harbor gang and the state. This new level emerged, we argue, because selection for trap limits was strong, but varied between regions facing different economic conditions. Since zones were created, they have adopted a set of cultural adaptations beneficial to the zone as a group, including legal trap limits and limited entry provisions, and have sought to resolve territorial disputes with neighboring zones.

We are reluctant, however, to suggest that the lobster zones are currently the dominant level of selection for lobstering practice for a few reasons. First, harbor gangs still exist, and may well adapt to the new organizational environment within the zones. Second, the superimposition of zone boundaries has had little short-term effect on the harbor gang territories. The reverse is not true, however, as zone boundaries were deliberately placed to coincide with existing informal boundaries (see Acheson 2003, pp. 102–103 for more detail). Third, the state Department of Marine Resources is strong and the possibility of top-down regulatory action is real. Moreover, zone councils have less autonomy than either informal gangs or the state and are legally constrained to rule on only a few topics. Therefore, while the lobster zones were produced by a new level of selection at the regional level, and that level appears to have been the dominant level of selection for a period of years, we do not assume that zones will adapt more nimbly than gangs, individuals or the state, in the future.

## Discussion and conclusion

This exercise has shown the multilevel selection framework (Waring et al. 2015) has been useful in an analytic capacity. Of course, evolutionary theory does not explain everything. To the contrary, in Maine, the economics of lobster fishing and defendability creates the underlying adaptive challenges to which cultural and social systems have responded. We have tried to understand that response from an evolutionary perspective.

A few useful lessons can be distilled from our structured case comparison. We have found that practices and institutions such as perimeter defense, trap limits, and limited entry rules constitute group-level cultural adaptations. Each group-level adaptation in our study appears to have solved a social dilemma in lobster management. This leads to our first lesson: social dilemmas in natural resource use pose adaptive challenges for groups of resource users, and solutions to these dilemmas are most likely to evolve in organizations that exist at corresponding social scale. This study also reveals the emergence of two new levels of social organization (gang, zone) over time, both of which also corresponded to major social dilemmas (territorial competition and trap escalation, respectively). Therefore, a second lesson is that if the stakes of a social dilemma are sufficiently high, new levels of social organization corresponding to that dilemma may emerge, as Waring et al. (2015) also noted.

So, when do conservation practices and institutions emerge? Nothing suggests that conservation solutions will always evolve via cultural group selection. In win–win cases such as the escape vent, where individuals benefit from a new conservation practice, there is no selection (and no need) for group-level solutions because there is no social dilemma. Additionally, if the conservation challenge is a social dilemma, a solution may never evolve unless conditions allow. With the benefit of hindsight, we can see which factors tend to predate the emergent group-level conservation solutions. Another lesson is that the stakes of success need to be sufficiently high to motivate cooperation and institutional change. This can be seen in the passage of both the double gauge and zone management laws, where major economic pressures and deadlock predated the final solutions. Another lesson is that a varied population of groups may facilitate group-level solutions. For instance, small, interdependent island communities tended to adopt cooperative behavior and enact innovative rules which then often spread among other gangs over time. Finally, group-level cultural evolution is more likely in a population of autonomous, persistent, economically interdependent, socially integrated groups with a shared fate. Moreover, a sedentary resource base makes defensive strategies viable. Where similar economic and social conditions are not met we might not expect the same degree of group level adaptation, and perhaps less emergent conservation. For example, state-level adaptations usually require state-level institutions such as the Department of Marine Resources to enact and uphold state-level adaptations and laws.

We have the benefit of observing aspects of the process of emergence as well. Cultural group selection requires a group level spread via migration, imitation, or group reproduction. So, we can ask which mechanisms of spread were most influential in lobstering. Although many of today’s lobstering practices emerged in the wake of group conflict, harbor gangs and lobster zones do not eliminate or subsume each other, nor do they typically split into daughter groups or go extinct. Fishermen only rarely migrate between harbor gangs. Thus, migration, group extinction, and group proliferation can be ruled out. We, therefore, conclude that the main mechanism of group selection in this context is between-group imitation and learning, even though we have few well substantiated cases of it. We suspect that research in other contexts will also find that imitative group selection generates resource conservation institutions more frequently than natural selection on groups via extinction and proliferation.

Our research is limited by our multiple factors. One gap is the lack of direct evidence of social learning, imitation, and social transmission between gangs and zones. Such evidence is often difficult to obtain, because social learning is ubiquitous, rapid, and rarely leaves a trace. Nonetheless, the transmission of practices and policies between groups is a key component of our explanation that requires more investigation. Also, would like to be able to test our conjectures more definitively, using more consistent data and more objective methods, but both are lacking. For example, in retrospect we can determine the dominant level of selection for a practice that was adopted and spread is the highest level for which selection was positive. While this is the simplest explanation for the emergence of the historical practice, we cannot make a forward prediction without more objective criteria and more complete data. Instead, we have developed a causal explanation which might be tested empirically in the future, or theoretically via simulation (e.g., Waring et al. 2017).

Looking forward, a prediction of the level at which adaptation in lobstering practice will be most robust could be useful in policy considerations. Is selection currently stronger on harbor gangs or lobster zones, or at another level entirely? Although the answer may determine the future of lobstering policy and practice, we are not well equipped to make such a prediction. Our analysis has identified historical shifts in the dominant level of selection, but future research might be designed to measure current status. Even imagining we did know the dominant level of selection with certainty, we may still not know the most pressing adaptive challenge facing social units at that level. We are certain, however, that cultural evolution among fishermen in groups such as harbor gangs and lobster zones has greatly facilitated the spread of cooperative territoriality and conservation practices and policies in the Maine lobster fishery.

## Footnotes

1 We use the terms “lobstermen” and “fishermen” to denote trade, not gender, in this article. Women who fish lobster are sometimes referred to as “female fisherman” (Van Allen 2014) and compose 4% of the lobstering population.

2 However, cultural group selection may also produce disadvantageous group cultural states, like a costly group fad.

3 Institutions, as complexes of coordinated and specialized individual behaviors, can emerge through individual behavioral optimization within a group (e.g., Smaldino 2014). While these within-group processes are important, we are concerned with the between-group process of institutional adoption, diffusion and competition.

4 Some group-beneficial individual traits are also individually beneficial (e.g., adopting commonly-used fishing terms). Such traits are often more likely to spread via individual-level selection.
